# Evaluating ATG Induction Therapy Outcomes After Commercial Kidney Transplantation: Insights from a Tertiary Hospital Experience

**DOI:** 10.3390/jcm14061896

**Published:** 2025-03-11

**Authors:** Sarah A. Albilal, Mohammed A. Gafar, Wesam S. Abdel-Razaq, Sarah Almugbil, Mohammed Alotaibi, Aiman A. Obaidat, Mohammad S. Shawaqfeh, Abdulkareem M. Albekairy

**Affiliations:** 1 King Abdulaziz Medical City, Ministry of National Guard Health Affairs, Riyadh 11426, Saudi Arabia; albilalsa@ngha.med.sa (S.A.A.); gafarmo@ngha.med.sa (M.A.G.); almugbilsa1@ngha.med.sa (S.A.); otaibim7@ngha.med.sa (M.A.); bekairya@ksau-hs.edu.sa (A.M.A.); 2 College of Pharmacy, King Saud bin Abdulaziz University for Health Sciences, Ministry of National Guard Health Affairs, Riyadh 14611, Saudi Arabia; razaqw@ksau-hs.edu.sa (W.S.A.-R.); obaidata@ksau-hs.edu.sa (A.A.O.); 3 King Abdullah International Medical Research Centre, Ministry of National Guard Health Affairs, Riyadh 11481, Saudi Arabia; 4 College of Medicine, King Saud bin Abdulaziz University for Health Sciences, Ministry of National Guard Health Affairs, Riyadh 14611, Saudi Arabia

**Keywords:** anti-thymocyte globulin, serum creatinine, kidney transplantation, clinical outcome, immunosuppressive therapy

## Abstract

**Background:** Kidney transplantation improves life expectancy in patients with end-stage renal disease but encounters ethical concerns, particularly in commercial transplantation, which yields worse outcomes. Anti-thymocyte globulin (ATG) is an immunosuppressant used as an induction therapy in transplantation. This study evaluates ATG induction therapeutic outcomes in commercial kidney transplants. **Methods:** A retrospective cohort analysis was conducted on adults who underwent commercial kidney transplantation and were subsequently admitted to King Abdulaziz Medical City spanning 2018 to 2023, with a follow-up period of one year. **Results:** A total of 70 commercial kidney transplant patients were evaluated by comparing patients who received ATG (n = 24) and those who did not (n = 46). ATG patients had elevated serum creatinine levels at admission (mean 457.5 vs. 172.6 µmol/L, *p* < 0.001). Over time, creatinine levels in the ATG group improved but remained higher than the non-ATG group (*p* < 0.001). Despite the higher early rejection rate in the ATG group, this difference was not statistically significant (*p*-value = 0.256). Elevated admission creatinine strongly predicted rejection (OR = 10.08, *p* < 0.001). **Conclusions:** Elevated admission creatinine is a significant predictor of rejection. Although the ATG group showed a higher early rejection rate, this difference was not statistically significant. Early rejection remains a concern, particularly within the first month after transplantation.

## 1. Background

Kidney transplantation is a pivotal treatment and is primarily indicated for patients with end-stage renal disease, providing improved long-term outcomes and quality of life compared to continued renal replacement therapy [1,2]. Economic disparities and inadequate healthcare services usually drive commercial kidney transplantation. This practice poses significant health risks and often involves exploiting vulnerable individuals, which raises several ethical and legal concerns. Commercial kidney transplantation is a practice in which people with end-stage renal disease (ESRD) pay a monetary value to receive a kidney transplant from a living unrelated donor [3]. Studies reveal worse medical outcomes and higher complication rates for commercial kidney recipients compared to legalized transplants [4]. Despite all efforts to suppress commercial kidney transplantation, unfortunately, it is a growing phenomenon [5]. Recent trends in the Middle East show an increased prevalence of organ transplants due to cultural factors and a shortage of organ donations [6]. At the World Health Organization’s (WHO) Second Global Consultation on Human Transplantation in March 2007, it was predicted that organ trafficking accounted for 5% to 10% of kidney transplants achieved annually throughout the world [7].

Commercial renal transplantation has emerged as an available outlet for expedited kidney transplantation due to the scarcity of organs, long waiting time for a deceased donor kidney offer, and lack of paired kidney donation programs in some transplant centers [3,5,8].

This practice is controversial, with some arguing that it is unethical to commercialize organ donation, while others argue that it provides a much-needed lifeline for people with ESRD [9]. In 2022, over a thousand commercial kidney transplants were performed worldwide. This number is expected to continue to grow in the coming years [10]. The most common adverse outcomes reported were short-term recipient complications. These include a variety of infections, surgical complications, recurrent hospitalizations, and typically a reduced kidney allograft survival rate compared to that of standard allografts [3,10].

The effective management of post-transplant immune responses is crucial to ensure graft survival and prevent further complications. Immunosuppressive drugs are utilized indispensably to prevent rejection, but careful therapeutic management is deemed necessary to balance the prevention of rejection while minimizing adverse effects, such as infections and malignancies [11]. Anti-thymocyte globulin (ATG) is a potent immunosuppressive treatment frequently used during the early phase of kidney transplantation to prevent acute graft rejection, particularly used as induction therapy (early post-transplantation) in high-risk kidney transplant recipients such as those with previous transplant failures [12,13]. By effectively depleting T-cells early on, ATG therapy allows for lower doses of maintenance immunosuppressive drugs (like calcineurin inhibitors), thus reducing their long-term toxicities [14].

In Saudi Arabia, the number of patients who have undergone commercial kidney transplantation abroad has grown dramatically. As a tertiary teaching hospital and one of the main solid organ transplant centers in the Kingdom of Saudi Arabia, we deal with commercial kidney transplantation very frequently [15]. All commercial kidney transplant patients arrive at our center’s emergency room for two days post-operation without documentation regarding the induction agent, the surgery details, or donor information. Effective management of post-transplant immune responses is crucial to ensure graft survival and prevent further complications. Immunosuppressive drugs are utilized indispensably to prevent rejection, but careful therapeutic management is deemed necessary to balance the prevention of rejection while minimizing adverse effects, such as infections and malignancies [16].

This study aims to evaluate the therapeutic outcomes of ATG induction therapy in patients who underwent commercial kidney transplants outside the Kingdom of Saudi Arabia. Furthermore, this study seeks to provide insights into the impact of ATG induction therapy that may enhance future treatment strategies, improve patient care, and guide decisions on using ATG in kidney transplantation.

## 2. Materials and Methods

### 2.1. Study Design

This retrospective cohort analysis examined all adult renal transplant patients who underwent commercial kidney transplants outside Saudi Arabia and were directly admitted to the nephrology transplant unit at King Abdulaziz Medical City (KAMC), Riyadh, Saudi Arabia, over the period from 2018 to 2023. Inclusion criteria included adult patients (≥18 years) with commercial kidney transplants and complete clinical records. Primary outcomes included one-year rates of rejection, infection, and graft or patient survival. Secondary outcomes included length of hospital stay, readmission rates, and surgical complications, if any. Demographic and baseline clinical characteristics were collected from electronic medical records of patients within one year after transplantation.

### 2.2. Data Management and Analysis

Data were statistically analyzed using the GraphPad Prism^®^ software package version 9.0 (San Diego, CA, USA), with outcome comparisons performed using appropriate statistical tests such as chi-square for categorical variables and non-parametric unpaired Student’s *t*-tests for continuous variables. Kaplan–Meier survival analysis was used to assess graft and patient survival with a stratified log-rank test at the two-sided significance level, while logistic regression was used to describe the association between the study endpoint (i.e., transplant rejection) and patient characteristics. We also used a confirmatory biopsy that is performed routinely and periodically to detect rejection early, before symptoms develop. All the tests were conducted using a two-tailed approach, and a *p*-value of less than 0.05 was deemed to indicate statistical significance.

Ethical approval was obtained from the Institutional Review Board at King Abdullah International Medical Research Center (KAIMRC) in Riyadh, Saudi Arabia, on 23 November 2023 (Approval Number: IRB/2940/23). Patient confidentiality was maintained by de-identifying all data.

## 3. Results

The study cohort included 70 adult patients who underwent commercial kidney transplants. Table 1 summarizes the general characteristics of the enrolled patients, stratified by those who received ATG (n = 24) and those who did not (non-ATG, n = 46). Most patients were male (75.0% in ATG vs. 78.3% in non-ATG) and aged 18–59 years. The gender distribution and age categories showed no significant differences between the groups. However, significant differences were observed in comorbid cardiovascular diseases, which were more frequent in the ATG group (66.7% vs. 41.3%, *p* = 0.044). The ATG group had markedly elevated serum creatinine levels at admission (with a mean of 457.5 µmol/L), which was statistically significantly higher than the non-ATG group (with a mean of 172.6 µmol/L, *p* < 0.001). Normal serum creatinine levels are defined as 64 to 114 µmol/L for men and 50 to 96 µmol/L for women. Elevated creatinine levels were notably prevalent in 95.8% of ATG patients, compared to 50.0% in the non-ATG group. Other variables, including infections, hospitalization length, and transplant rejection, showed no significant differences. Mortality within one year was minimal and comparable (4.2% in ATG vs. 2.2% in non-ATG, *p* = 0.635).

**Figure 1:**
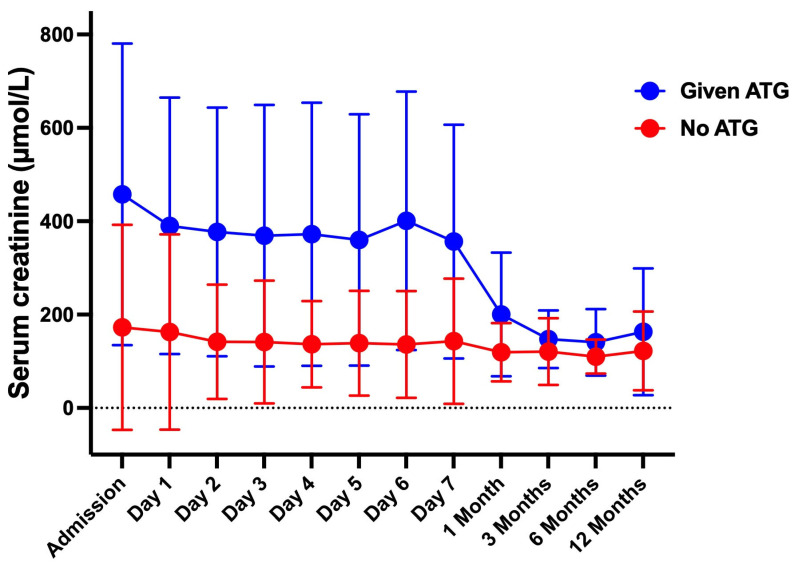
Serum creatinine levels (measured in µmol/L) over time in two groups of patients: those given ATG (blue line) and those not given ATG (red line). Statistical significances measured using multiple non-parametric unpaired *t*-tests and ordinary two-way ANOVA with main effect only.

Figure 1 illustrates the chronological (time-based) changes in serum creatinine levels for the ATG and non-ATG groups. While the creatinine levels in the non-ATG group remained consistently lower throughout the follow-up period, there was a slight but not statistically significant decrease over time (*p* = 0.175). At admission, the ATG group had significantly elevated serum creatinine levels compared to the non-ATG group (*p* < 0.001). Creatinine levels in the ATG group did not change substantially during the initial 7-day follow-up period (*p* = 0.298). However, the ATG group displayed prominent and significant reductions in creatinine levels one month after admission (*p* = 0.001). Despite showing improvement, the creatinine levels in the ATG group remained significantly higher than in the non-ATG group from admission through the first month post-transplant (*p* < 0.001). Nonetheless, over time, the creatinine levels in the ATG group gradually and steadily declined from the 7th-day mark to 3 months, and continued to decrease until the end of the follow-up period (*p* = 0.014 and 0.009, respectively). Thereafter, creatinine levels were comparable between the two groups (*p* = 0.159 and 0.169, respectively).

**Figure 2:**
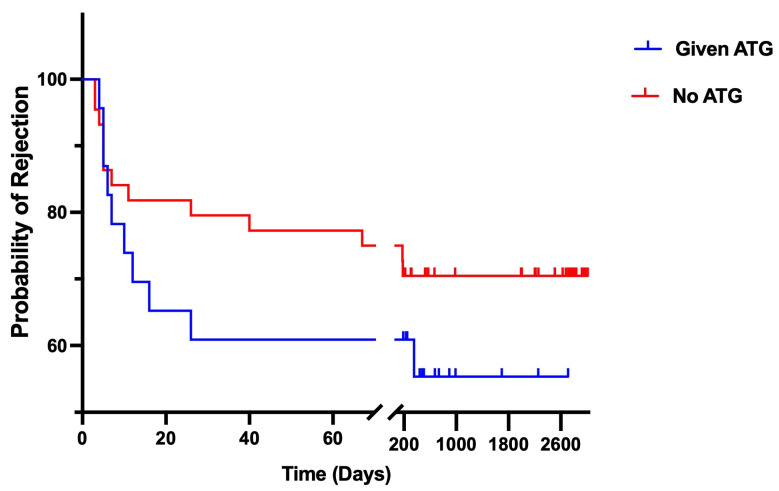
The Kaplan–Meier survival analysis for the composite endpoint of transplant rejection and death, stratified by the inclusion of AGT. The median survival rate for both groups could not be determined since neither curve reached a 50% probability of survival/rejection by the final time point on the survival curve.

Kaplan–Meier survival analysis examined transplant rejection and mortality over one year, as illustrated in Figure 2. Three patients were excluded from the analysis because their rejection occurred within two days of admission. Both groups initially showed sharply rising rejection rates. Most rejections occurred within the first month (90.0% of total rejections in the ATG-treated group vs. 71.4% in the non-ATG group). This highlights a pronounced early rejection pattern in both groups. By the end of the follow-up period, the 1-year survival rate was higher in the ATG group (44.7%) compared to the non-ATG group (29.5%). However, the log-rank test found no statistically significant difference (*p* = 0.256), with a hazard ratio of 1.60 [95% CI: 0.67–3.82]. These findings may suggest that while ATG therapy may have some benefit in reducing long-term rejection rates, its impact on survival and rejection is not conclusive.

Finally, Table 2 presents the logistic regression analysis of participant variables with the study endpoint, which was defined as renal transplant rejection, to explore predictors of transplant rejection. Elevated serum creatinine at admission was strongly associated with rejection (odds ratio = 10.08, *p* < 0.001). Other factors, including gender, age, BMI, comorbidities, infections, and previous transplant history, showed no statistically significant association with rejection outcomes.

## 4. Discussion

ATGs are biological polyclonal depleting agents that have been employed as an induction treatment to induce prophylactic immunosuppression in kidney transplant recipients [15]. Such powerful agents have been claimed to lower graft rejection rates [16] and allow early withdrawal of steroids, as well as enable early hospital discharge [17]. The Kidney Disease Improving Global Outcomes (KDIGO) Guidelines in 2009 recommended using potent T-cell-depleting agents for patients at a high immunological risk, as supported by several clinical studies [18].

As shown in Table 1 of our results, investigating different variables between the two study groups indicated no significant differences except for the serum creatinine levels, which were markedly elevated in the ATG group. However, after one month, the level of serum creatinine gradually declined in the ATG group and became insignificantly different between the two groups thereafter. Several studies have explored the long-term outcomes of ATG compared to other immunosuppressive agents [19,20,21]. When analyzed compared to interleukin-2 receptor (IL-2R) antagonists as an induction therapy against acute rejection in kidney transplant patients, significantly lower serum creatinine levels were observed in IL-2R-treated patients [22]. In contrast, another study comparing ATG to corticosteroid therapy found no significant difference in creatinine levels between the study groups over a period of 12 months after transplantation [23]. However, ATG-administered patients were found to experience more pronounced and significant adverse effects [12,24]. While ATG may prevent acute rejection, patients may develop additional CMV infections and malignancies compared to those treated with IL-2R antagonists [25]. A study by Marghoob et al. (2019) investigated the change in serum creatinine levels during a follow-up period after ATG induction therapy, reporting initially slightly elevated levels followed by a gradual decline over a period of three months [26]. Additionally, their study revealed that 11.4% of the patients experienced acute rejection with a mean time of 9.61 days post-transplantation, along with an additional 2.03 days of hospitalization compared to the control group. This can be consistent with our findings, which showed that 90% of the ATG group experienced rejection within one month after transplantation.

The current results suggest that elevated serum creatinine at admission in the ATG group may be significantly associated with graft rejection. However, other studies have shown that induction treatment with ATG provided no additional benefit regarding graft function or rejection compared to standard induction with other agents [27,28]. A recent study from Korea that compared the outcomes of induction therapies found no superiority of ATG over basiliximab in terms of delayed graft function or rejection and concluded that the type of induction regimen had no significant impact on graft failure or patient survival [29]. Furthermore, several meta-analyses of IL-2R antagonist induction therapy indicated no increased risk of graft rejection or all-cause mortality [30,31,32,33].

This study has some limitations, including its retrospective design and small sample size. Due to its retrospective nature, several confounding factors and variables related not only to the recipient patients but also to the donors were not measured. Moreover, the study was unable to account for variability in commercial transplant practices because of the bias inherent in its retrospective design. The nature of commercial transplant practice is complicated, with social, legal, and ethical concerns that made data collection/ verification difficult. Nevertheless, the study offered valuable insights into the correlation between elevated serum creatinine levels in the ATG-treated group and the acute rejection rate, aligning with other published reports. It also supports findings from other studies indicating that ATG did not demonstrate superiority over other induction treatments. Further research is recommended to systematically explore the relationship between serum creatinine levels, eGFR, and ATG induction therapy compared to other induction treatments. Moreover, a valid comparison between commercial and non-commercial transplant may provide a better insight, and a cost-effectiveness study is warranted.

## 5. Conclusions

This study found that patients who received ATG induction had higher initial serum creatinine levels, likely indicating advanced severity or early rejection. However, these levels substantially decreased over time and became comparable to patients not receiving ATG. Early rejection rates were higher in the ATG group, albeit not significantly. Elevated serum creatinine at admission may serve as a reliable predictor of rejection, highlighting the need for further research to optimize ATG usage and improve long-term transplant outcomes. The single center experience with such scares rather than growing population may shed a light on necessity to unify the practice guidelines across centers.

## Figures and Tables

**Table 1:** General characteristics of adult renal transplant patients who underwent commercial kidney transplants; n = 70.

Variable	Given ATG (n = 24)	No ATG (n = 46)	*p*-Value ^†^
Gender, n (%) Male Female	18 (75.0%) 6 (25.0%)	36 (78.3%) 10 (21.7%)	0.758
Current age (in years), n (%) 18–39 40–59 ≥60	14 (58.3%) 5 (20.8%) 5 (20.8%)	18 (39.1%) 18 (39.1%) 10 (21.7%)	0.236
BMI, n (%) <18.5 (underweight) 18.5–24.9 (healthy weight) 25–29.9 (overweight) ≥30 (obesity)	0 (0.0%) 10 (41.7%) 4 (16.7%) 10 (41.7%)	1 (2.2%) 11 (23.9%) 17 (37.0%) 17 (37.0%)	0.176
Primary cause of kidney disease, n (%) Renal-related diseases (GN, ADPKD) Other diseases (DM, HTN, others) Unknown ^¶^	1 (4.2%) 8 (33.3%) 15 (62.5%)	4 (8.7%) 26 (56.5%) 16 (34.8%)	0.861
Comorbidities, n (%) ‡ Cardiovascular diseases Diabetes mellitus Hypertension Dyslipidemia	16 (66.7%) 11 (45.8%) 20 (83.3%) 4 (16.7%)	19 (41.3%) 19 (41.3%) 35 (76.1%) 7 (15.2%)	0.044 0.716 0.483 0.874
Dialysis type, n (%) Hemodialysis Peritoneal Dialysis	22 (100.0%) 0 (0.0%)	41 (91.1%) 4 (8.9%)	NA
Missing data (n = 3) ^¶^			
Kidney transplantation, n (%) First time Second time	23 (95.8%) 1 (4.2%)	44 (95.7%) 2 (4.3%)	0.972
History of viral hepatitis, n (%) None Any hepatitis strain	23 (95.8%) 1 (4.2%)	42 (91.3%) 4 (8.7%)	0.485
History of Tuberculosis, n (%) No Yes	21 (87.5%) 3 (12.5%)	45 (97.8%) 1 (2.2%)	0.077
Serum creatinine level (on admission) Mean ± (SD) Median (range, IQR)	457.5 ± (323.0) 336.5 (114–1194, 583.3)	172.6 ± (219.7) 116.5 (50–1313, 75.0)	< 0.001
Serum creatinine level (on admission) Normal ^#^ Elevated	1 (4.2%) 23 (95.8%)	23 (50.0%) 23 (50.0%)	< 0.001
Viral infections No Yes (both CMV and BK)	4 (16.7%) 20 (83.3%)	10 (21.7%) 36 (78.3%)	0.615
Bacterial infections No Yes (both UTI and Bac)	13 (54.2%) 11 (45.8%)	18 (39.1%) 28 (60.9%)	0.229
Length of stay (hospitalization) ^§^ Mean ± (SD) Median (range, IQR)	15.7 ± (12.5) 12 (2–40, 21.0)	12.2 ± (13.1) 9 (1–84, 10.8)	0.285
Transplant rejection, n (%) No Yes	14 (58.3%) 10 (41.7%)	32 (69.6%) 14 (30.4%)	0.347
Type of rejection, n (%) ^Ψ^ Acute TCMR Acute ABMR Mixed type	6 (60.0%) 2 (20.0%) 2 (20.0%)	11 (78.6%) 0 (0.0%) 3 (21.4%)	0.213
Rejection post-transplantation, n (%) Within 1 month 1–3 months 3–6 months 6–12 months	9 (90.0%) 0 (0.0%) 0 (0.0%) 1 (10.0%)	10 (71.4%) 2 (14.3%) 2 (14.3%) 0 (0.0%)	0.211
Frequency of 1-year readmission, n (%) None 1–2 episodes ≥3 episodes	5 (20.8%) 13 (54.2%) 6 (25.0%)	14 (30.4%) 22 (47.8%) 10 (21.7%)	0.692
Reasons for 1-year readmission, n (%) ^Φ^ Infections Transplant rejection Surgical Complications Others	9 (47.4%) 10 (52.6%) 12 (63.2%) 2 (10.5%)	22 (68.8%) 11 (34.4%) 15 (46.9%) 2 (6.3%)	0.484
Death within one year, n (%) No Yes	23 (95.8%) 1 (4.2%)	45 (97.8%) 1 (2.2%)	0.635

^†^ Calculated using the chi-square/Fisher exact test for categorical variables or the unpaired *t*-test for continuous variables. ^‡^ The percentages were calculated from the total number of patients in the respective group. ^¶^ Unknown or missing data were excluded from the statistical calculations. ^#^ Normal serum creatinine levels are defined as 64 to 114 µmol/L for men and 50 to 96 µmol/L for women. ^§^ Following follow-up hospital admission after a commercial kidney transplant. ^Ψ^ The percentages were calculated from the total number of rejections within the respective group. ^Φ^ The percentages were calculated from the total number of readmissions within the respective group. Abbreviations: GN (Glomerulonephritis); ADPKD (Autosomal dominant polycystic kidney disease); DM (diabetes mellitus); HTN (Hypertension); CMV (Cytomegalovirus); BK (BK virus, also known as human polyomavirus); UTI (urinary tract infection); Bac (Bacteremia); TCMR (T-cell–mediated rejection); ABMR (antibody-mediated rejection).

**Table 2:** Logistic regression analysis of patient variables versus study endpoint; n = 70.

Variable	Study Endpoint ^†^		Odds Ratios (95% CI)	*p*-Value (2-Tailed)
	Negative (Controlled) (n = 46)	Positive (Rejection) (n = 24)		
Gender Males (n = 54) Females (n = 16)	35 (76.1%) 11 (23.9%)	19 (79.2%) 5 (20.8%)	0.84 (0.25 to 2.77)	0.771
Age at diagnosis Less than 60 years (n = 55) 60 years and older (n = 15)	37 (80.4%) 9 (19.6%)	18 (75.0%) 6 (25.0%)	1.37 (0.42 to 4.44)	0.600
BMI <25 Healthy (n = 22) ≥25 Overweight and Obese (n = 48)	14 (30.4%) 32 (69.6%)	8 (33.3%) 16 (66.7%)	0.88 (0.30 to 2.52)	0.804
Comorbidity None (n = 10) Yes (n = 60)	6 (13.0%) 40 (87.0%)	4 (16.7%) 20 (83.3%)	0.75 (0.19 to 2.96)	0.682
Kidney transplants First time (n = 67) Second time (n = 3)	44 (95.7%) 2 (4.3%)	23 (95.8%) 1 (4.2%)	0.96 (0.08 to 11.12)	0.972
Serum creatinine level (admission) Normal (n = 24) Elevated (n = 46)	22 (47.8%) 24 (52.2%)	2 (8.3%) 22 (91.7%)	10.08 (2.12 to 47.93)	0.004
Viral infections (CMV and BKV) No (n = 57) Yes (n = 13)	37 (80.4%) 9 (19.6%)	20 (83.3%) 4 (16.7%)	0.82 (0.22 to 3.01)	0.767
Bacterial infections (UTIs and Bac) No (n = 43) Yes (n = 27)	26 (56.5%) 20 (43.5%)	17 (70.8%) 7 (29.2%)	0.54 (0.19 to 1.54)	0.246

^†^ The study endpoint was defined as renal transplant rejection. Negative endpoints indicate patients whose kidney function was controlled. Abbreviations: CMV (Cytomegalovirus); BKV (BK virus, a member of the polyomavirus family); UTIs (urinary tract infections); Bac (Bacteremia).

## Data Availability

The data presented in this study are available on request from the corresponding author due to privacy reasons.
